# *Cytobacillus* sp. Strain HMBC3 from Saudi Arabian Soil Degrades Low-Density Polyethylene

**DOI:** 10.4014/jmb.2409.09023

**Published:** 2024-12-06

**Authors:** Narjes J. Alamer, Munirah. F. Aldayel, Ashraf Khalifa

**Affiliations:** Biological Science Department, College of Science, King Faisal University; P.O. Box 400, Al-Ahsa 31982, Saudi Arabia

**Keywords:** Biodegradation, *Cytobacillus*, soil, Saudi Arabia, plastic, pollution

## Abstract

Considering the dangerous effects on the environment of commonly used packaging materials like low-density polyethylene (LDPE), finding a practical and environmentally safe method for decomposing plastics is crucial. In this study, a bacterial strain (HMBC3) was identified in plastic-contaminated soil samples from eastern Saudi Arabia and showed potential for breaking down LDPE, as revealed by results from weight loss, Fourier-transform infrared spectroscopy (FTIR), scanning electron microscopy (SEM), and gas chromatography-mass spectrometry (GC-MS). HMBC3 was discovered among bacterial isolates in a mineral nutritional medium (MNM) enhanced with LDPE as the only carbon and energy source. The strain caused changes in the media pH from 7.0 ± 0.03 to 6.23 ± 0.05, while the LDPE also showed 20.4% weight loss. In addition, the 16S rRNA gene similarities revealed a 98.9% homology between HMBC3 and *Cytobacillus* sp., indicating their close similarity. The high efficiency of strain HMBC3 in biodegrading plastic could potentially lead to its widespread development as an eco-friendly way to eliminate or minimize environmental plastic pollution.

## Introduction

Plastics have become a fundamental requirement in every aspect of our lives, replacing earlier substitutes like metal, glass, and wood, with several advantages [1]. Numerous products employ plastic, including clothing, furniture, building materials, and car parts. Polymers are more affordable, have superior technological properties, and are lighter in weight [2]. The world produces 0.15 billion tons of synthetic polymers annually and with a 12%annual increase in plastic production worldwide, experts predict that the pace of plastic buildup in the environment will reach 25 million tons [3]. In 2019, packaging and building & construction became Europe's largest end-use markets for, plastics, with the automotive industry coming in third, according to the Plastics Europe organization [4]. Also in 2019, Asia accounted for 45% of plastic production worldwide, and Europe was next with 25% [5]. The packaging sector is one of the most significant end markets for plastic in the Middle East, including Saudi Arabia. Experts anticipate the plastic packaging market to grow from USD 5.82 billion in 2020 to USD 7.92 billion by 2026 [6, 7]. Furthermore, in 2019, 10.2% of plastics were imported [4]. Saudi Arabia, with an annual production of over six million tons of plastic, ranks as one of the largest producers of plastic waste worldwide, accounting for around 15% of the nation's total municipal waste [8].

Polyethylene is a man-made polymer and the most widely used synthetic polymer in industry [9]. Low-density polyethylene (LDPE) bags are used around the world 500 billion to 1 trillion times a year. This huge production and use of the material causes it to build up in the environment. Polyethylene (PE) is resistant to natural degradation because it has strong C-H and C-C bonds, so not only is it among the main sources of pollution, getting rid of it is also a big problem [10]. Accumulated plastic is disposed of by uncontrolled burning and landfilling, which releases toxins back into the air and causes a number of health problems. Burning plastic also releases harmful substances that may cause human carcinogens, immune system disorders, and respiratory ailments [11]. The durability of LDPE leads to long-lasting persistence in the environment, posing challenges for waste management, especially when compared to other plastics like high-density polyethylene (HDPE) and polypropylene (PP), which have different degradation rates and environmental impacts. Research into LDPE degradation is crucial, as it may reveal microbial strains or enzymes capable of breaking it down, thereby offering bioremediation solutions. Recently, scientists are focusing on the biodegradation of LDPE wastes. Microbial biodegradation of LDPE plastic waste is one of the feasible strategies to resolve its harmful accumulation [12].

Degradation is a natural process by which microbes break down complex organic materials into simpler ones while obtaining nutrients. Microbes can survive thanks to the energy and carbon sources found in plastic garbage. Polymers have the ability to decompose in both aerobic and anaerobic environments, providing a significant natural cleanup for toxic waste disposal sites [13, 14]. The first step in biodegradation is biodeterioration. This is when microbial oxidative enzymes free the carbonyl groups from the linear carbon chain. Next, oxidation lowers the carbonyl groups, and carboxylic acid groups form. The second step is biofragmentation, where enzymes secreted by microorganisms hydrolyze and fragment polymer carbon chains, releasing intermediate products. The third step is bioassimilation, in which bacteria or fungi absorb and metabolize the small hydrocarbon fragments generated by biofragmentation. The final step is mineralization, which is an intracellular conversion that involves the release of carbon dioxide and water as hydrolyzed products turn into microbial biomass [15]. However, treating the polymer with heat, chemicals, or ultraviolet (UV) light before it is used can help it oxidize and create a C=O group. This can speed up the first step in biodegradation and shorten the time it takes to break down [16]. Any alteration in the properties of polymers, including their molecular weight, mechanical strength, and surface features, is known as biodegradation. In other words, it refers to the process of breaking down a substance into smaller pieces by microbial feeding and biochemical transformation [17]. Researchers have conducted several studies to isolate plastic-degrading bacteria from soil. Researchers discovered a significant diversity in the isolated bacterial species, as well as in their capacity to degrade various types of plastic. *Pseudomonas* and *Bacillus* were the most isolated genera, followed by *Staphylococcus* sp. and other species [18]. Due to its large molecular weight and hydrophobic nature, LDPE has demonstrated resistance to decomposition; however, reports indicate that a number of microbes can degrade LDPE plastic [10]. Recent research has revealed that microorganisms such as *Pseudomonas aeruginosa* [19], *Brevibacillus* spp., *Aneurinibacillus* spp. [20], *Pseudomonas fluorescens* [21], *Bacillus cereus*, *Brevibacillus borstelensis* [22], *Enterobacter* spp. [23], *Streptomyces coelicoflavus* [24], and *Serratia* sp. [25] can degrade LDPE.

Microorganisms can depolymerize these polymers by secreting enzymes that break them down into monomers, which can then enter cell walls and serve as energy sources [26]. Microorganisms produce many intracellular and extracellular enzymes, including multicopper oxidase, manganese peroxidase, laccase, alkane hydroxylase, alkane monooxygenase, and ligninolytic enzyme [27].

Researchers have not extensively studied the microorganisms that can remove plastic pollution from the environment, including the eastern area of the Kingdom of Saudi Arabia. In the Eastern Province of Saudi Arabia, researchers have found that the bacteria *Ochrobactrum* sp., *Pseudomonas citronellolis*, *P. aeruginosa*, and *Cupriavidus taiwanensis* degrade pyrene and phenanthrene [28]. The selection of the Arabian region for bacterial isolation is supported not only by the unique environmental conditions but also by the prevalence of LDPE pollution, making it a relevant site for our research. In particular, plastic contamination in the Al-Ahsa region poses risks to human health and other living organisms across various industrial, medical, and domestic settings. These circumstances make it imperative to restore the ecosystem to a healthy, original state. Therefore, our aim was to investigate the bacterial species responsible for LDPE degradation and characterize them using phenotypic and genotypic traits. To this end, soil samples taken from a plastic landfill were used to isolate and identify bacterial species capable of breaking down LDPE. Weight loss analysis and FTIR measurements of the morphological changes in the plastic powder were used to quantify the degree of degradation. By using 16S RNA gene analysis, the molecular identification of the isolated bacterial strains was also established.

## Materials and Methods

### Sample Collection and Study Area

Soil samples were collected from Half Moon Bay (37° 27' 48.7872'' N and 122° 25' 42.9096'' W) in the eastern region of Saudi Arabia. The soil samples were taken at varying depths of approximately 10-15 cm, and about 1 g of soil was aseptically transported in a sterile Ziploc plastic bag within 24 h to the King Faisal University laboratory in Al-Ahsa [29]. The physical parameters measured were a temperature of 24°C, and pH of 7.69 ± 0.04.

### Inoculum Preparation

To isolate the bacteria, 1 g of soil sample was carefully mixed with 50 ml of a 0.85% NaCl solution, which was prepared by dissolving 8.5 g of NaCl in 1 L of distilled water and subsequently autoclaved to ensure sterility. This saline solution provides an isotonic environment that helps maintain the integrity of bacterial cells during the isolation process. After mixing, the inoculated solution was incubated at 30°C for 3 h at 150 rpm. This shaking facilitates the fractionation of bacterial cells from the soil particles, allowing for better dispersion of the bacteria in the solution. Then, a serial dilution method was employed to further isolate the bacterial cells. The diluted samples were then subjected to the spread plate technique using nutrient agar (NA) [30]. To make the inoculum adapt to the MSM, we incubated the pure colony with MSM for one week using the following ingredients in one liter of distilled water: 0.1% (NH_4_)_2_SO_4_, 0.1% NaNO_3_, 0.1% K_2_HPO_4_, 0.1% KCl, 0.02% MgSO_4_, 0.001% yeast extract, and 2 g LDPE powder [10]. To obtain a pure colony, the spread plate technique was applied, and the plate was incubated at 30°C for three days [31]. The isolated bacteria were maintained in Nutrient Broth (NB) with 20%glycerol and kept at -20°C for preservation. The isolates were streaked on NA slants and kept at 8°C for normal usage. These strains were then subcultured routinely, every 3-4 weeks.

### Characterization and Identification of Bacterial Isolates

After growing on NA plates at 30°C for three days, the morphological characteristics of the bacteria isolates, including their colony shape, color, size, and elevation, were identified. The gram response was similarly calculated using the aforementioned approach [32].

Further identification of the bacterial strains was done using Analytical Profile Index 20E (API) as a biochemical identification technique, and it was applied according to the instructions of Biomérieux (France), the manufacturer,

First, InstaGene Matrix (Bio-Rad, USA) was used for DNA extraction from the isolated bacteria. To precipitate the cells, we heated them at 95°C for 5 min, followed by centrifugation 10,000 ×*g* for 10 min [33]. A 12.5 μl PCR Master Kit (HiMedia, India), 1 μl of each primer, and 1 μl of the DNA template were used in a 25 μl volume for the PCR reaction. The negative control was left out. The universal primers, forward primer (8-F) 5'-AGAGTTTGATCCTGGCTCAG-3' and reverse primer (1492R) 5'-GGTTACCTTGTTACGACTT-3', were used to amplify the 16S rRNA gene [34]. Following initial denaturation at 94°C for 5 min, the major PCR stages of denaturation were carried out at 94°C for 45 s, annealing at 55°C for 60 s, extension at 72°C for 60 s, and final extension at 72°C for 10 min. After amplification, the PCR product was resolved by electrophoresis in a 1% agarose gel using 100 ml of 1x TAE buffer. EtBr was injected at a concentration of around 2-4 L because it binds to DNA and may be observed on a gel documentation system [35].

Next, we employed an automated DNA sequencing device (model 3730XL, Applied Biosystems, USA) to sequence the 16S rRNA gene for bacterial isolates, and then used the BLAST algorithm to compare the results with those in the GenBank database (National Center for Biotechnology Information, USA). The Mega7 program was used to create phylogenetic trees using the neighbor-joining technique based on the TamuraNei model.

### Biodegradation Efficiency

Pure colonies were incubated for six months in MSM [36] with 2 g of LDPE powder at 30°C and 100 rpm to evaluate the efficiency of the isolated bacteria in degrading LDPE.

### Media pH Values and Weight Loss for LDPE Powder

The survival and enzymatic activity of bacteria depend heavily on pH, which influences the overall biodegradation rate, so the efficiency of HMBC3 to degrade LDPE plastic was studied on MNM. The pH value was estimated at the beginning and at the end of the experiment using a pH meter [37].

The LDPE powder after 6 months of incubation was removed by filtration, rinsed with sterile distilled water, and left to dry for three days [38]. The weight loss percentage was determined [39], using:

$$Weight\;loss\;\%=\frac{initial\;weight-final\;weight}{initial\;weight}\times 100.$$

### FTIR Analysis

Changes in the structure of plastic powder following incubation with bacteria were analyzed using Equinox 55 FTIR (Bruker, UK) [40]. After 180 days of incubation in the synthetic medium (MSM), the LDPE powder was separated from the media using filter paper, rinsed with sterile distilled water, and then dried at room temperature for three days. This washing step is essential to remove any residual microbial biomass or contaminants that could interfere with the spectral analysis. The surface structure of the LDPE was examined using the Equinox 55 FTIR spectrometer (Bruker Optik GmbH, USA), following the methodology outlined by Pramila and Ramesh (2011). For each LDPE sample, a spectrum was recorded over the wavelength range of 400 to 4,000 cm^-1^, at a resolution of 2cm^-1^, with 32 scans. These spectra were then compared with the control powder that was incubated in Mineral Salts Medium without bacteria.

### Gas Chromatographic Analysis (GC-MS)

The LDPE degradation products of the bacterial isolates were examined using GC-MS. After six months of incubating LDPE in MSM, the media were centrifuged at 8,000 ×*g* for 5 min at room temperature, followed by filtration. Diethyl ether was used as a solvent to obtain the degradation products. Then, 10 ml of MSM was dissolved in 10 ml of diethyl ether and left overnight before being separated using a separating funnel [41]. A gas chromatography-mass spectrometer (GCMS-QP2010 SE, Shimadzu, Japan) was used to analyze the organic extract. It had an Rxi-5Sil capillary column that was 30 mm long, 0.25 mm wide, and 0.25 m thick [42]. The mobile phase was powered by helium, which flowed at a rate of 1 ml/min. Precisely 1 μl of sample was fed at 220°C in splitless mode into the split/splitless inlet. The oven temperature was programmed to start at 50°C for 5 min before being increased by 7°C/min to 240°C for 15 min in the GC-MS technique analysis. The ion source's temperature was 220°C, whereas the contact was 250°C. The data were collected using the 50–550 amu scan mode [43].

### Scanning Electron Microscopy (SEM)

SEM analysis was used to examine the morphological changes on the surface of polyethylene after incubation with bacterial isolates. Polyethylene powder was recovered from the MSM medium after incubation for 6 months. Samples were fixed with 2.5% glutaraldehyde in 0.05 M cacodylate buffer (90 min at 4°C) [44]. The dehydrated samples were sputter-coated with a gold layer (Edwards S150B, UK) [44]. The surface was investigated using a Philips-X LP30 scanning electron microscope (Netherlands). The sputtering was achieved after passing the pure and dry argon gas in the coating chamber, under a vacuum. The plate voltage was 2000 volts, and the current flow was 15 mA. A thickness of 2 nm of gold was achieved during a sputtering time of 10 s [44]. The sample was then examined under SEM.

## Results

The objective of our study was to identify and characterize bacterial species from Saudi Arabia's eastern region that have the potential to break down LDPE plastic. Out of 44 bacteria isolated from a soil sample from Half Moon Bay, Saudi Arabia, only HMBC3 bacteria were purified and identified as HMBC3 by using the enrichment process on a synthetic medium with LDPE as the only source of carbon and energy (Table 1). The physical parameters were pH 7.69 ± 0.04 and a temperature of 24°C (Table 1).

### Characterization and Identification of Bacterial Isolates

**Fig. 1:**
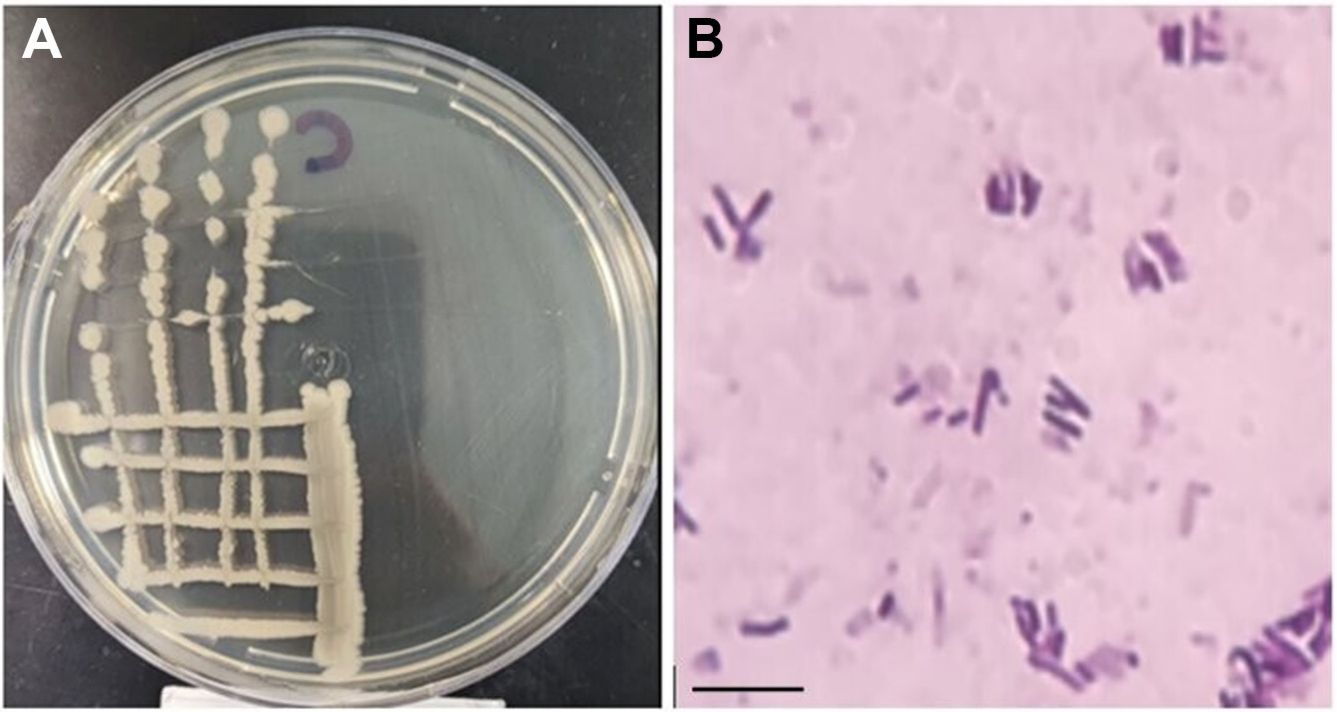
Colonies of HMBC3 bacteria on nutrient agar plates (A) and under the microscope (B). Magnification was 100×; bar = 500 μm.

One bacterium, strain HMBC3, was obtained out of the enrichment technique and found to be efficient in degrading LDPE. Fig. 1 and Table 2 present the morphological characteristics. The isolate formed oval-shaped colonies with undiluted margins and raised elevations. The colonies had a diameter of 5-8 mm and were made of faint mud (Table 2).

### API Biochemical Test

In the biochemical identification for HMBC3 (Table 3), five out of twenty tests were positive. The test results showed the decarboxylation of arginine by arginine dihydrolase (ADH), the formation of the gelatinase enzyme (GEL), the fermentation of hexose sugars glucose and mannose (GLC and MAN), and the fermentation of the disaccharide sucrose (SAC). On the other hand, the negative results included ONPG, LDC, ODC, CIT, H2C, URE, TDA, IND, VP, INO, SOR, RHA, MEL, AMY, and ARA. ONPG: O-nitrophenyl-b-D-galactopyranoside.\ ADH: Arginine dihydrolase amino acid. LDC: Lysine decarboxylase. ODC: Ornithine decarboxylase. CIT: Citrate.\ H2S: hydrogen sulfide. URE: Urease.\ TDA: Tryptophan deaminase. IND: Indole tryptophan.\ VP: the Voges-Proskauer test. GEL: gelatinase enzyme. GLC: fermentation of glucose. MAN: fermentation of mannose. INO: fermentation of inositol. SOR: fermentation of sorbitol. RHA: fermentation of rhamnose. SAC: fermentation of sucrose. MEL: fermentation of melibiose. AMY: fermentation of amygdalin. ARA: fermentation of arabinose.

### Evolutionary Relationships

**Fig. 2:**
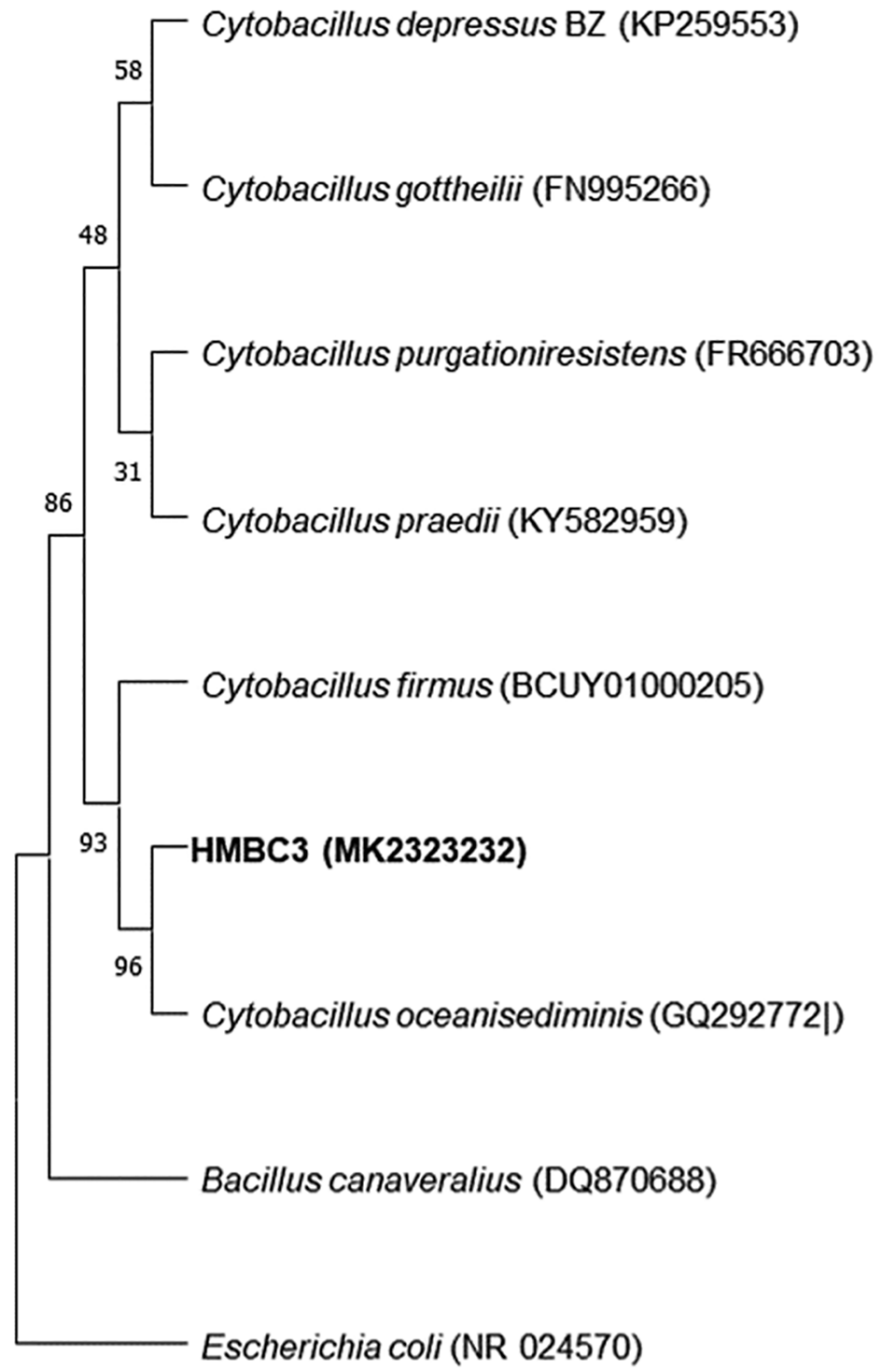
The evolutionary phylogenetic history for the identified isolate species was inferred using neighborjoining method. The optimal tree is shown. The percentage of replicate trees in which the associated taxa clustered together in the bootstrap test (100 replicates) is shown next to the branches.

The evolutionary history was inferred using the neighbor-joining method [45]. The maximum composite likelihood method [46] computed the evolutionary distances in Fig. 2, which represent the number of base substitutions per site. This analysis involved 18 nucleotide sequences. Codon positions included were 1^st^+2^nd^+3^rd^+Noncoding. All ambiguous positions were removed for each sequence pair (pairwise deletion option). There were a total of 1,500 positions in the final dataset. Evolutionary analyses were conducted in MEGA11 [47].

Based on 16S rRNA gene similarities, HMBC3 was found to be most closely related to the genus *Cytobacillus*. HMBC3 showed 98.9% homology with *Cytobacillus oceanisediminis* (Accession No. GQ292772), forming a monophyletic group (Table 2, Fig. 2), while *Escherichia coli* (NR024570) formed a distinct outgroup (Table 2 and Fig. 2).

### Biodegradation Efficiency

**pH Values of the Media and the Weight Loss for LDPE Powder**

A pH meter was used to measure the changes in the MNM pH before and after being incubated with LDPE. Table 4 shows that after six months of incubation, the pH dropped from 7.0 ± 0.03 to 6.23 ± 0.05 (Table 4). The pH value of untreated control media remained constant.

### Weight Loss Measurements

After 6 months of incubation with HMBC3, the dry weight reduction of LDPE powder was measured (Table 3). The LDPE weight significantly decreased by 20.4%, whereas the untreated reference powder's dry weight only slightly changed (1.7%).

### FTIR Analysis

**Fig. 3:**
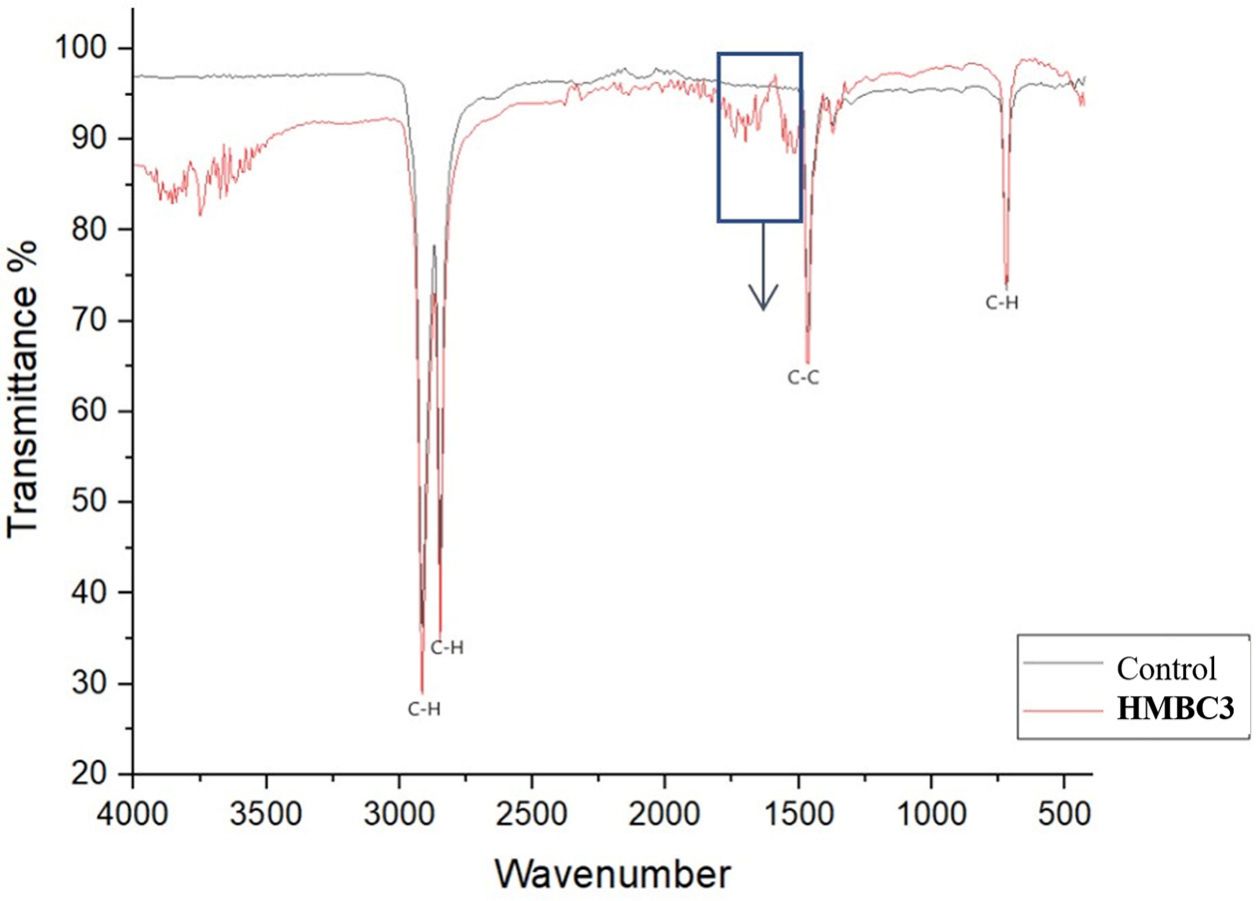
FTIR spectra of LDPE powder after incubation for 6 months with HMBC3 and control.

To understand the biodegradation process, the carbonyl groups or double bonds in LDPE were analyzed using FTIR to detect changes (Fig. 3). The Control revealed a variety of peaks showing the complexity of the LDPE, as typical absorption bands were assigned at C-H mono-bending (715 cm^-1^), C-C stretching (1,461 cm^-1^), C-H stretching 2,847 cm^-1^, and 2,914 cm^-1^.

**Fig. 4:**
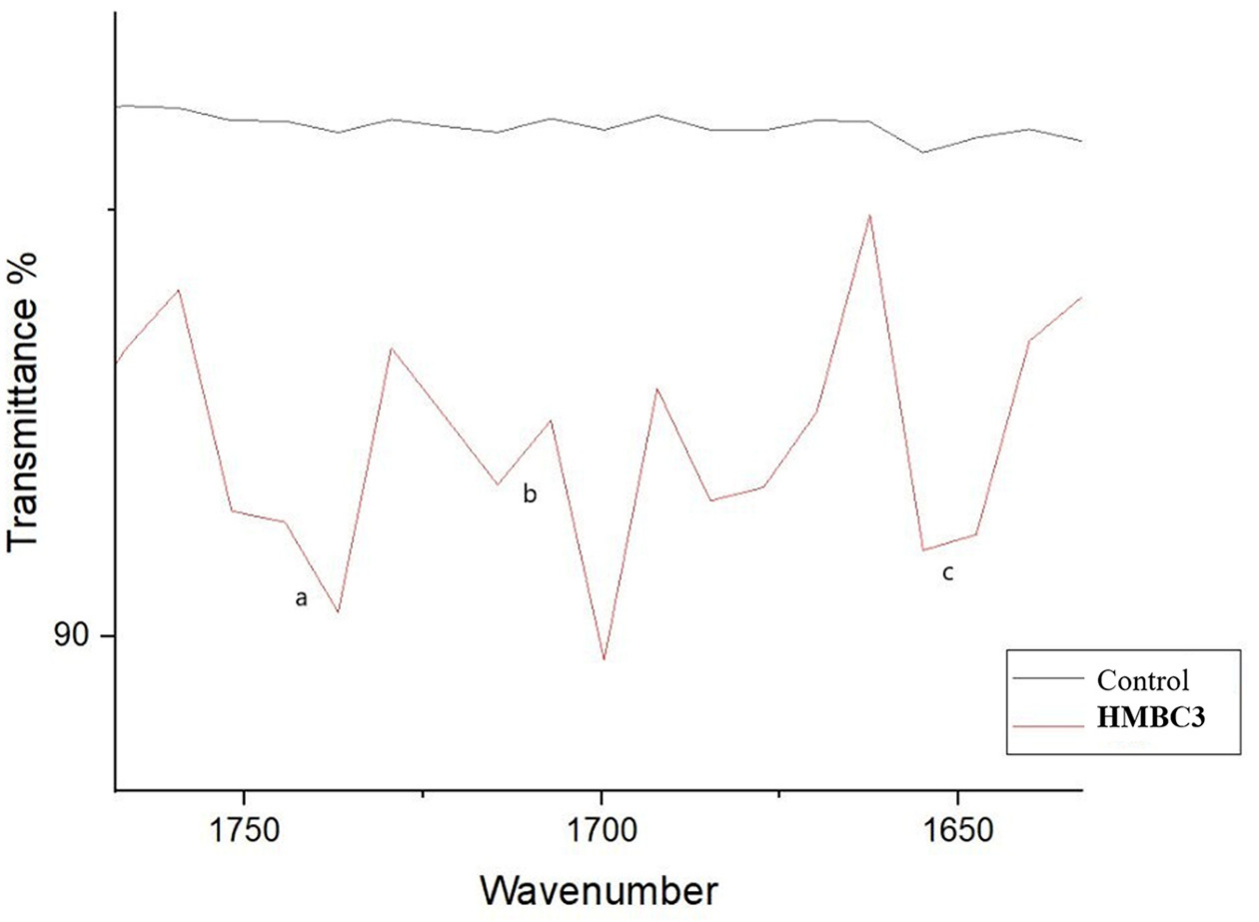
FT-IR spectra of LDPE powder after 6 months incubation with HMBC3 and control. (**A**) ketones (1,740 cm^-1^). (**B**) acids (1,715 cm^-1^), and (**C**) double bonds (1,640 cm^-1^). Fig. 4. A magnified view of the boxed area in Fig. 3.

The FTIR showed increase in the intensity of peaks 1,461 cm^-1^, 2,847 cm^-1^, and 2,914 cm^-1^ in the LDPE incubated with HMBC3. Also, formation of typical double bonds are represented by the peak (1,650 cm^-1^), formation of acids (1,715 cm^-1^), and ketone production (1,740 cm^-1^) (Fig. 4).

### GS-MS Analysis

**Fig. 5:**
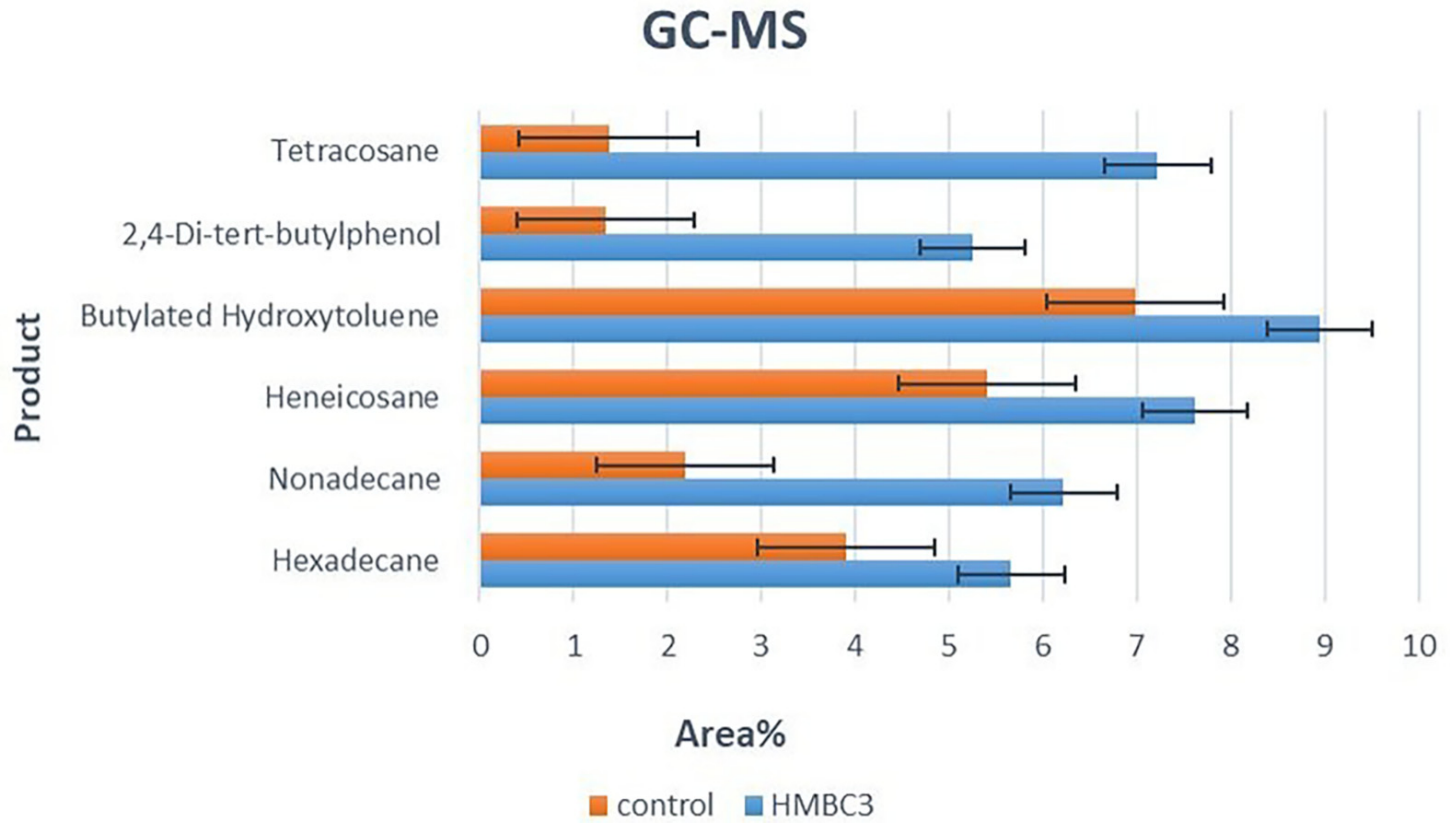
GC-MS for the highest degradable compounds; Hexadecane, Nonadecane, Heneicosane, Butylated Hydroxytoluene, 2,4-Di-tert-butylphenol, Tetracosane after 6 months incubation with HMBC3, and control.

GC-MS analysis was used to identify various substances in the LDPE-treated sample, including volatile and semi-volatile alkanes, aromatic hydrocarbons, esters, and both saturated and unsaturated fatty acids (Fig. 5 and Table 5). After six months of incubation with HMBC3, 37 biodegraded polyethylene compounds were detected, with key products such as hexadecane, nonadecane, heneicosane, butylated hydroxytoluene, 2,4-di-tert-butylphenol, and tetracosane being identified as the most degradable. The GC-MS chromatogram revealed a diverse array of compounds in both the HMBC3 strain and the control, with 33 compounds identified in the HMBC3 sample and 37 in the control, indicating a rich chemical profile resulting from the incubation process. Notably, the dominant products in the HMBC3 sample included heneicosane, hexadecane, 2,4-di-tert-butylphenol, butylated hydroxytoluene, nonadecane, and tetracosane, suggesting significant microbial metabolic activity associated with the degradation of LDPE.

### SEM

**Fig. 6:**
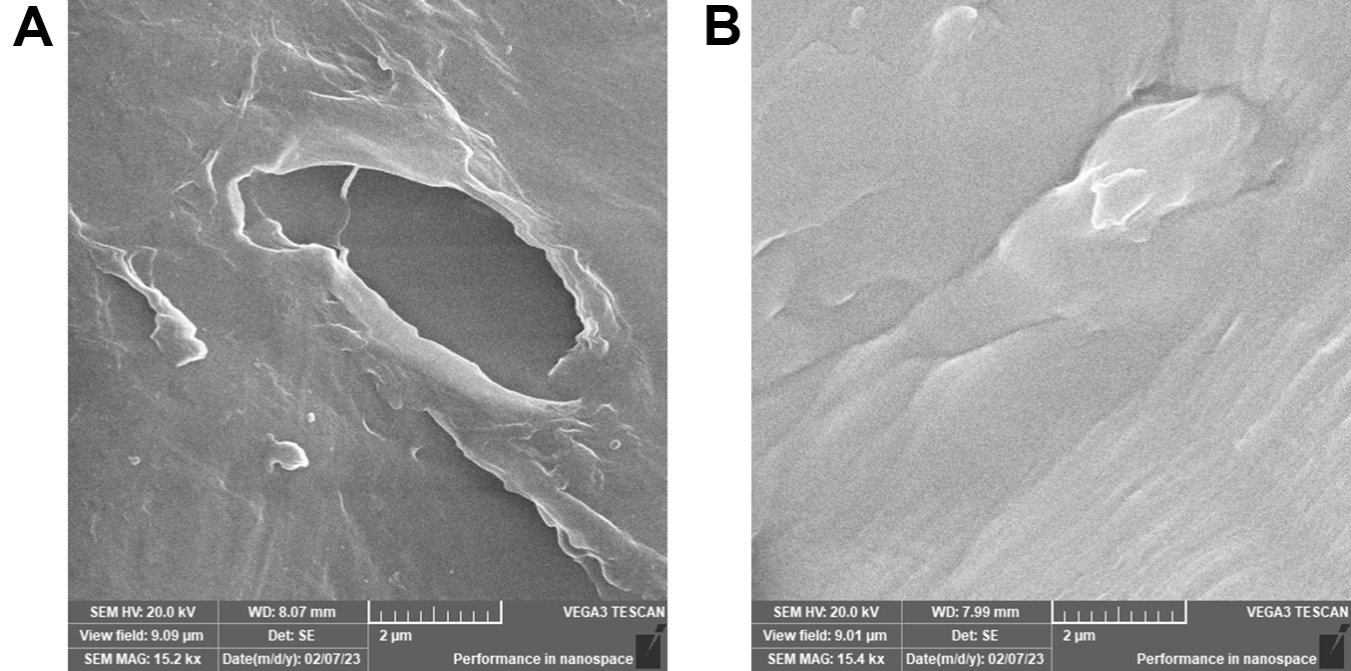
LDPE under SEM, after 6 months incubation; (A) HMBC3, (B) control.

The first step for bacteria in destroying LDPE is by colonizing the surfaces and penetrating them; these processes can be examined by SEM analysis. After 6 months of incubation, the treated LDPE showed physical modifications and surface cracks as a result of bacteria adhering to and colonizing the LDPE surface (Fig. 6). Under the same circumstances, the control provided a smooth surface with no cracks, and no particles adhered to the surface.

## Discussion

Our study aimed to isolate and characterize bacterial species from the eastern region of Saudi Arabia with the capacity to degrade LDPE plastic from a soil sample from Half Moon Bay, Saudi Arabia. LDPE powder was added to mineral media (MNM) as the only carbon source. The medium's pH changed after 6 months of incubation, and the LDPE's weight was reduced. FTIR, GC-MS, and SEM tests all showed that the HMBC3 bacteria from the soil had the ability to break down LDPE. HMBC3 was versatile in its ability to utilize the biochemical metabolites of API 20E strips.

The 16S ribosomal RNA gene, widely recognized as a powerful tool for identifying bacteria at the genus and species levels, serves as a basis for phylogenetic inferences across prokaryotes [48]. Based on the similarities in their 16S rRNA gene sequences, we discovered that HMBC3 shares the closest relationship with the genus *Cytobacillus*, exhibiting a homology of 99.48%. These findings provide further evidence of the 16S rRNA's efficiency in identifying bacterial species.

The biochemical identification by API 20E showed positive results for ADH, GEL, GLU, MAN, and SAC. This *Cytobacillus* strain HMBC3 bears a striking resemblance to [49], where *Bacillus mycoides*, isolated from pipe water in Lagos Metropolis, demonstrated positive results for ONPG, ADH, CIT, VP, GLU, GEL, and SAC. *Bacillus thuringiensis* isolated from Egyptian soils had positive results in only ADH, TDA, and VP tests [50]. These findings indicate variability among bacterial species and strains with respect to the consumption of the metabolites contained in the API 20E.

The biochemical identification results obtained through the API 20E system indicate that the strain exhibits key metabolic activities relevant to the degradation of LDPE. The decarboxylation of arginine by arginine dihydrolase (ADH) suggests a capacity for utilizing amino acids, which can be beneficial in nutrient-limited environments, potentially enhancing the strain's resilience and adaptability. The formation of gelatinase (GEL) indicates the ability to break down proteins and could contribute to the degradation of complex organic materials that may accompany LDPE in the environment. Furthermore, the fermentation of hexose sugars like glucose and mannose (GLC and MAN), along with sucrose (SAC), demonstrates the strain's ability to utilize various carbon sources for energy. This metabolic flexibility is crucial as it allows HMBC3 to thrive in diverse habitats and may facilitate co-metabolism with LDPE, enhancing degradation efficiency. In comparison to previously reported LDPE-degrading strains, these biochemical characteristics suggest that our strain possesses a robust metabolic profile, potentially allowing it to degrade LDPE more effectively. The combination of enzymatic activities and fermentation capabilities may play a significant role in breaking down LDPE and utilizing its components, ultimately contributing to the strain's overall effectiveness in bioremediation efforts. Recent studies support these findings; for instance, *Pseudomonas aeruginosa* and *Bacillus cereus* have demonstrated LDPE degradation potential and positive reactions to the production of ADH and GEL [49]. Similarly, Kučić *et al*. [50] used API 20E to characterize LDPE-degrading *Bacillus* species and *Pseudomonas alcaligenes*, underscoring the system's utility in identifying metabolic functions critical for plastic biodegradation.

The fact that the pH of the MNM that was inoculated with HMBC3 decreased shows that *Cytobacillus* can use LDPE as a carbon source and start to use it metabolically during incubation. After 180 days of incubation, researchers such as Biki, S.P., *et al*. [36] reported similar findings, showing that the pH of the LDPE-containing media decreased from 7.12 to 6.67 for *Ralstonia* sp. and from 7.12 to 7.03 for *Bacillus* sp. Additionally, Das and Kumar [53] demonstrated that *Bacillus amyloliquefaciens* lowers the pH from 7.0 to 5.11 after 60 days of LDPE incubation. It is likely that the *Cytobacillus* action on LDPE caused the release of several intermediates, including acids, which could reduce the medium's pH level. Extracellular enzymes and the metabolic activity of microorganisms using LDPE as a carbon source have been considered as explanations for the pH decrease. Furthermore, LDPE depolymerization and fragmentation would result in products such as fatty acids and organic acids, causing the pH to fall [44, 54].

A considerable amount of data suggests that the weight of LDPE decreases as it degrades. According to several studies, weight loss is the first sign of polythene decomposition. The results, which were in agreement with other investigations, revealed a considerable decrease in LDPE weight at 20.4% as a result of *Cytobacillus* activity. It has been recently reported that the novel species *Bacillus tropicus* demonstrated a significant level of LDPE degradation, resulting in a weight loss of 10.15% after 40 days of incubation, while *Bacillus subtilis* and *Bacillus licheniformis* showed weight losses of 3.49% and 2.83%, respectively [55]. This result aligns with previous findings that weight loss correlates with the microbial breakdown of LDPE, but HMBC3 shows a higher degradation rate compared to *Bacillus tropicus* and *Bacillus subtilis*. Additionally, bacteria such as *Serratia* sp., *Stenotrophomonas* sp., and *Pseudomonas* spp. have demonstrated varying degrees of LDPE degradation, with *Serratia* sp. achieving the highest 40% weight loss after 150 days, and *Stenotrophomonas* sp. and *Pseudomonas* sp. showing 32% and 21%weight loss, respectively [56]. These comparisons reveal that HMBC3 performs similarly to *Pseudomonas* sp., but less effectively than *Serratia* sp. and *Stenotrophomonas* sp., suggesting that further optimization or co-culturing may enhance its degradation capacity. These results underscore the variable efficiency of microbial strains in degrading LDPE and support the use of diverse strains for bioremediation purposes.

To use the carbon and energy they contain, microorganisms release a range of enzymes that first break these complex compounds down into smaller building blocks [60]. The result is a reduction in the weight of the LDPE [57]. pH and weight loss demonstrated the ability of both isolates to use LDPE as their exclusive carbon source.

Because it is sensitive to molecular structures, FTIR is widely used to analyze macromolecule interactions during LDPE degradation. This is due to FTIR's sensitivity to molecular settings [60]. FTIR analysis tracked the structural transition of LDPE, which leads to polymer degradation as well as other physical and chemical processes that impact the material's quality [1]. FTIR was used to evaluate the structural changes of the LDPE to further monitor the biodegradability of the HMBC3 [60]. FTIR analysis serves as the method to assess changes in polyethene bonds and chemical composition. FTIR was used to confirm that LDPE incubated with HMBC3 showed changes in the original peaks as well as the appearance of new peaks. Ketones (1,740 cm^-1^) in LDPE created the new peaks. Acids (1,715 cm^-1^), double bonds, and b (1,640 cm^-1^) were also identified. This suggests that various chemical intermediates emerged during the biodegradation process. A related LDPE spectrum profile was found by [55]. They found two new peaks at 918 cm^−1^ and 3,375 cm^−1^, which are linked to the O-H bond and acidic groups in *Bacillus tropicus* [58]. Substituting an oxygen atom on a long C-C bond for a hydrogen atom can produce functional groups like ester groups, carbonyl groups, and ether groups [61]. Researchers have found that alkane hydroxylases play a crucial role in aerobic alkane degradation, hydroxylating C-C bonds to generate primary and secondary alcohols [62]. Furthermore, microbial alkene monooxygenase epoxidizes alkenes during the biodegradation of LDPE, producing ether as a product [63]. These results suggest that degrading microorganisms can identify structural changes in LDPE through changes in peak location, the emergence of new peaks, or the elimination of existing ones.

GC-MS was used to confirm the biodegradation of LDPE further. The GC-MS analysis highlights a variety of compounds identified in HMBC3-treated and control samples after six months of incubation. The most reliable method for identifying volatile degradation products is GC-MS [61], which revealed the presence of several key intermediates of LDPE degradation, such as alkanes (*e.g.*, hexadecane, pentadecane, nonadecane, heneicosane) and high-molecular-weight hydrocarbons (*e.g.*, tetracosane, tetracontane). These compounds result from partial breakdown of the polymer chains, indicating that the LDPE structure has undergone scission and oxidation processes. Additionally, butylated hydroxytoluene and 2,4-di-tert-butylphenol were identified as degradation products that may facilitate LDPE degradation by preventing polymer cross-linking and enhancing the breakdown of the polymer chains [59-62].

In the HMBC3-treated sample, compounds such as alcohols (*e.g.*, 1-Tridecanol), acids (*e.g.*, n-Hexadecanoic acid), and esters (*e.g.*, Octanoic acid, decyl ester) were exclusively observed, suggesting more advanced stages of degradation as these functional groups are typical of microbial activity and oxidative processes. Compounds like 3,5,5-Trimethyl-2-cyclohexen-1-one indicate further oxidation of cyclic hydrocarbons, a step closer to full mineralization. In contrast, the control sample mainly contains unbranched alkanes and high-molecular-weight hydrocarbons with fewer oxidation products, implying less advanced degradation. The presence of unique compounds in the control, such as 4-Ethylheptane, Cyclohexene, and 1-methyl-4-(1-methylethenyl)-, which are absent in the HMBC3-treated sample, suggests that HMBC3 treatment may facilitate the breakdown of LDPE, leading to a broader range of degradation intermediates and accelerating the degradation process. The presence of these compounds has been shown to facilitate LDPE degradation, as demonstrated in previous studies [59-62]. The findings underscore the effectiveness of HMBC3 in enhancing degradation pathways and producing intermediate compounds that represent progressive LDPE breakdown.

Generally speaking, the GC-MS patterns for *Aspergillus nomius* and *Streptomyces* sp. [12], as well as for *Bacillus licheniformis* SARR1 [63], are comparable to ours. Nevertheless, the chemicals discovered may be common or exclusive to the microbial group.

Moreover, different microbial groups' actions resulted in a variety of polyethylene-biodegraded chemicals. For instance, after 90 days of incubation with *Aspergillus nomius* and *Streptomyces* sp., we found six and eight compounds, respectively [12]. Based on our findings and those of other researchers, it can be concluded that the degree of LDPE breakdown varies based on several factors, including the microbial strain and the conditions of growth and degradation. Studies done recently have shown that *Cytobacillus* S19, a strain of bacteria found in contaminated soil in China, can break down petroleum oil very well because it makes surfactants [61], and *Bacillus subtilis* can break down LDPE [64-67]. After treating the LDPE with HMBC3 to create certain grooves, cavities, or bioerosion on its surface, SEM was carried out to determine the variation in the surface caused by the enzymatic depolymerization process. A similar result was observed by [59] after a 40-day incubation with *Bacillus tropicus*.

The pH of the control sample remains unchanged, indicating the absence of microbial activity. However, the control sample showed a modest weight loss, and the GC-MS analysis detected LDPE-degrading substances, albeit in smaller quantities than HMBC3, suggesting a likely abiotic degradation. The SEM data provided further confirmation of *Cytobacillus*'s breakdown of LDPE without any pretreatment. Our findings appear to open the door for further research to determine the particular process by which *Cytobacillus* break down LDPE.

## Conclusion

In this work, we found an LDPE-degrading bacterial strain (HMBC3) that was related to *Cytobacillus* with homology of 98.9%. The strains' capacity to break down LDPE plastic was demonstrated by a decrease in media pH, weight loss, FTIR structural alterations, SEM, and the generation of GCMS intermediates. For the purpose of reducing or eliminating LDPE contaminants from the environment, *Cytobacillus* can be employed widely.

## Supplemental Materials

Supplementary data for this paper are available on-line only at http://jmb.or.kr.



## Figures and Tables

**Table 1:** The sample location, temperature, and pH values for the bacterial isolates.

No.	Site	Nature	Location	Depth (cm)	Temperature (°C)	pH	Bacterial isolate
1	Half Moon Bay	soil	26.198972, 50.029222, 26.198774, 50.029494	10-15	24	7.69±0.04	HMBC3

**Table 2:** HMBC3 morphological characteristics and gram reaction.

Morphological characteristics						
Strain	Shape	Color	Margin	Diameter (mm)	Elevation	Gram reaction
HMBC3	Oval	Faint mud	Entire	5-8 mm	Raised	+
Pairwise Similarity (%)						
Name	Strain	Accession	Pairwise Similarity (%)			
*Cytobacillus* sp.	HMBC3	OQ606968	98.94%			

**Table 3:** Characteristics of HMBC3 using API 20E.

HMBC3									
OPNG	ADH	LDC	ODC	CIT	H2S	URE	TDA	IND	VP
-	+	-	-	-	-	-	-	-	-
GEL	GLU	MAN	INO	SOR	RHA	SAC	MEL	AMY	ARA
+	weak +	weak +	-	-	-	weak +	-	-	-

ONPG: O-nitrophenyl-b-D-galactopyranoside.\ ADH: Arginine dihydrolase amino acid. LDC: Lysine decarboxylase. ODC: Ornithine decarboxylase. CIT: Citrate.\ H2S: hydrogen sulfide. URE: Urease.\ TDA: Tryptophan deaminase. IND: Indole tryptophan.\ VP: the Voges-Proskauer test. GEL: gelatinase enzyme. GLU: fermentation of glucose. MAN: fermentation of mannose. INO: fermentation of inositol. SOR: fermentation of sorbitol. RHA: fermentation of rhamnose. SAC: fermentation of sucrose. MEL: fermentation of melibiose. AMY: fermentation of amygdalin. ARA: fermentation of arabinose.

**Table 4:** Changes in the media's pH levels and a percentage reduction in LDPE weight.

Sample	pH for SM before incubation	pH for SM after six months of incubation	Reduction % in LDPE powder weight
HMBC3	7.0 ± 0.03	6.23 ± 0.05	20.4%
Control	7.0 ± 0.03	7.0 ± 0.04	1.7%

**Table 5:** Compounds identified in GC/MS analysis of HMBC3 and control samples after six months of incubation.

Number	Compound name	HMBC3	Control
1	2,4-Dimethylhexane	+	+
2	4-Ethylheptane	-	+
3	3,3-Dimethyloctane	+	+
4	Cyclohexene, 1-methyl-4-(1 methylethenyl)-	-	+
5	5-Methylundecane		+
6	4,6-Dimethyldodecane	+	+
7	2,6,10-Trimethyldodecane	-	+
8	3,5,5-Trimethyl-2-cyclohexen-1-one	+	+
9	Tridecane	+	+
10	2-Methyldecane	-	+
11	4-Methyldodecane	+	+
12	Tetradecane	+	+
13	2,6,11-Trimethyldodecane	+	+
14	Pentadecane	+	+
15	Hexadecane	+	+
16	Heptadecane	+	+
17	Nonadecane	+	+
18	Eicosane	+	+
19	Heneicosane	+	+
20	2-Methyltridecane	-	+
21	Butylated Hydroxytoluene	+	+
22	Phenol, 2,4-bis-(1,1-dimethylethyl)	+	+
23	Hexadecane, 2,6,10,15-tetramethyl	+	+
24	Octacosane	-	+
25	Silane, trichlorooctadecyl-	-	+
26	Dotriacontane	+	+
27	n-Triacontane	+	+
28	Tetracontane	+	+
29	Tetratetracontane	+	+
30	Tetrapentacontane	+	+
31	3,3-Dimethyloctane	+	-
32	2,6-Dimethylundecane	+	-
33	4,6-Dimethyldodecane	+	-
34	1-Tridecanol	+	-
35	Docosane	+	-
36	Tetracosane	+	-
37	Octanoic acid, decyl ester	+	-
38	Phthalic acid, ditridecyl ester	+	-
39	n-Hexadecanoic acid	+	-
40	1,2,3,4-Tetramethylbenzene	-	+
41	3-Methyldecane	-	+
42	Hexane, 3,3-dimethyl-	-	+
43	2-Butyl-1-octanol	-	+
44	1-Dodecanol	-	+
45	Octadecane	+	+
46	1-Decanol, 2-hexyl-	+	+
Identified Compounds		33	37
Unidentified compound	Not detected in the database	6	2

+: indicates that the compound is present, and – indicates that the compound is absent.
