# Supplementary figures and images for: Cytobacillus sp. Strain HMBC3 from Saudi Arabian Soil Degrades Low-Density Polyethylene
Source: J Microbiol Biotechnol. 2024 Dec 6;35:e2409023. doi: 10.4014/jmb.2409.09023 (PMC11876017; doi:10.4014/jmb.2409.09023)

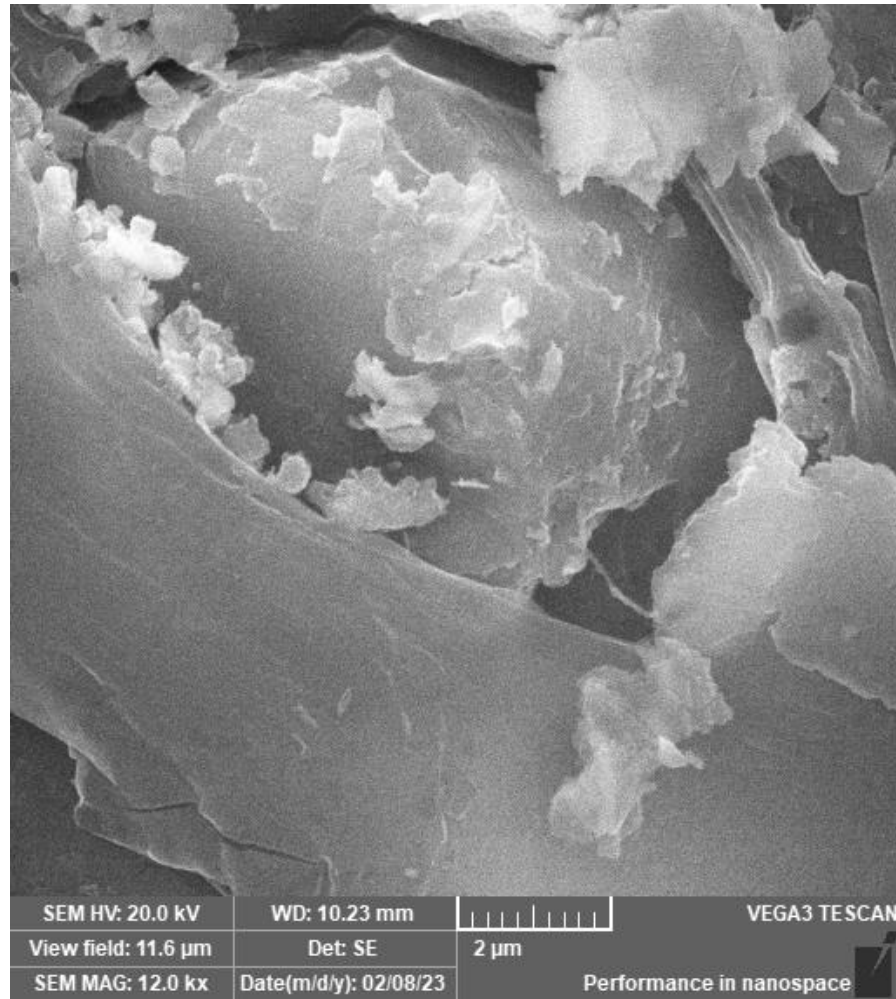

Fig. S1. LDPE under SEM, after 6 months incubation for HMBC3.

Supplement: Supplementary file 1 [file jmb-35-e2409023-supple.pdf]
